# A Population-Based Retrospective Study of Biliary Tract Cancers in Alberta, Canada

**DOI:** 10.3390/curroncol28010044

**Published:** 2021-01-13

**Authors:** Carissa Beaulieu, Arthur Lui, Dimas Yusuf, Zainab Abdelaziz, Brock Randolph, Eugene Batuyong, Sunita Ghosh, Oliver F. Bathe, Vincent Tam, Jennifer L. Spratlin

**Affiliations:** 1 Department of Medicine, University of Alberta, Edmonton, AB T6G 2R3, Canada; 2 Cross Cancer Institute, University of Alberta, Edmonton, AB T6G 1Z2, Canada; ag_lui@yahoo.com (A.L.); droncoz@live.com (Z.A.); sunita.ghosh@ahs.ca (S.G.); jennifer.spratlin@ahs.ca (J.L.S.); 3 Southern Philippines Medical Center, Davao City 8000, Philippines; 4 Delta Hospital, Delta, BC V4K 3V6, Canada; dimas.yusuf@alumni.ubc.ca; 5 Department of Clinical Oncology, Kasr Al-Aini School of Medicine, Cairo 11562, Egypt; 6 Department of Family Medicine, University of Alberta, Edmonton, AB T6G 1K4, Canada; brandolph@ualberta.ca; 7 Tom Baker Cancer Centre, University of Calgary, Calgary, AB T2N 4N2, Canada; eugene.batuyong@ahs.ca (E.B.); oliver.bathe@ahs.ca (O.F.B.); vincent.tam@ahs.ca (V.T.); 8 Department of Surgery, University of Calgary, Calgary, AB T2N 2T9, Canada; 9 Department of Oncology, University of Alberta, Edmonton, AB T6G 1Z2, Canada

**Keywords:** biliary tract neoplasms, cholangiocarcinoma, gallbladder cancer, survival outcomes, chemotherapy

## Abstract

**Background:** Biliary tract cancers (BTC) are uncommon malignancies and are underrepresented in the literature. **Methods:** We performed a retrospective population-based review of adult patients with biopsy-confirmed BTC in Alberta from 2000 to 2015. Demographic data, risk factors, symptoms, treatment, and staging data were collected and analyzed. Survival analyses were completed. **Results:** A total of 1604 patients were included in our study, of which 766 (47.8%) were male. The median age at diagnosis was 68 (range 19–99). There were 374 (23.3%) patients with resectable tumors at diagnosis versus 597 (37.2%) with unresectable tumors. Of the patients, 380 (21.5%) received chemotherapy (CT) and 81 (5.0%) underwent radiation therapy. There was a clear trend with worsening stage and performance status associated with shorter median overall survival (OS). Ampulla of Vater tumors had the best median OS (25.69 months), while intrahepatic bile duct cancers had the worst (5.78 months). First-line palliative CT regimens included gemcitabine+cisplatin (OS 14.98 months (mo), *n* = 212), single agent gemcitabine (OS 12.42 mo, *n* = 22), capecitabine (OS 8.12 mo, *n* = 8), and capecitabine+gemcitabine (OS 6.93 mo, *n* = 13). Patients with advanced or metastatic disease who received first-line gemcitabine+cisplatin had a median OS of 11.8 months (*n* = 119). **Conclusion:** BTCs have poor survival. Worse outcomes occur in higher stage and poorer Eastern Cooperative Oncology Group (ECOG) performance status patients across all tumor subtypes. Tumor resectability at diagnosis was associated with better OS. Our study supports the use of gemcitabine+cisplatin as a combination first-line palliative CT, as patients treated in Alberta have a comparable OS to that reported in the ABC-02 phase III study.

## 1. Introduction

Biliary tract cancers (BTC) are a rare group of malignancies comprising of, in decreasing incidence, gallbladder cancers (GBCs), cholangiocarcinomas arising from intra- or extrahepatic ducts (IHC or EHC), and ampulla of Vater tumors (AVT) [1]. Generally, BTCs present with more advanced disease as many are asymptomatic in early stages and, as a result, have a poor prognosis [2]. Cholangiocarcinomas constitute only 3% of all gastrointestinal malignancies [3,4]. Data suggest an increase in the incidence of IHC tumors in the United States over the past 20 years [5,6], which could be partially attributed to an increase in the prevalence of risk factors for intrahepatic disease, such as liver cirrhosis, hepatitis C infection, or alcohol-related liver disease [5]. GBCs, unlike other BTCs, are more common in women, likely due to the increased prevalence of gallstone disease among this population [7]. However, while infection, inflammation, and irritation have been postulated as risk factors, cholelithiasis has the most significant association with the disease [8,9].

There are limited trials investigating BTC management [10]. If resectable, BTCs are curable, and surgery is therefore the best option for nonmetastatic disease [3,4,11]. Post-resection survival is generally poor, although cure rates are higher among those with pathologic negative margins [3]. The BILCAP trial is the only randomized study to date confirming the benefit of adjuvant chemotherapy (CT) post-resection in early-stage BTCs. BILCAP determined that 6 months of adjuvant capecitabine improved median overall survival (OS) compared to observation alone (51 vs. 36 months, *p* < 0.01, HR0.71) [12].

Unresectable or metastatic disease is approached with palliative intent and has poor OS [10]. Options for therapy include biliary drainage or stent placement, radiation therapy, and systemic CT [3,4]. The limited evidence available in this heterogenous population suggests that fluoropyrimidine- or gemcitabine-based therapies show response rates of 0–41% and 8–36%, respectively [10]. Standard of care systemic CT for locally advanced or metastatic biliary tract tumors worldwide is based on the ABC-02 randomized phase III clinical trial, which included over 400 patients with locally advanced or metastatic disease. In this patient population, OS was improved with cisplatin and gemcitabine compared to gemcitabine alone (11.7 vs. 8.1 months, *P* < 0.001, respectively) [1].

The latest body of research encompassing BTC focuses on earlier diagnosis, less invasive testing, and more targeted therapy. The analysis of cellular components such as circulating tumor DNA (ctDNA) has garnered interest for playing a potential role in earlier and less invasive methods of diagnosis, genomic profiling, and therapeutic response monitoring in BTC [13]. Over the last decade, the identification of key molecular pathways and genetic markers in BTC has identified prospective targeted therapies. For example, aberrancies noted in the fibroblast growth factor (FGF) pathway in intrahepatic cholangiocarcinoma procured phase I and II trials using FGFR inhibitors with promising safety and efficacy data [14].

The purpose of our study was to collect descriptive data on patients with BTC to better understand our Alberta population’s profile. Furthermore, we set out to determine the survival outcomes between different treatment regimens and provide more insight on the treatment being offered for BTC in Alberta, Canada, which represents the Western patient population well.

## 2. Methods

Approval from the Health Research Ethics Board of Alberta (HREBA) was obtained to perform a retrospective population-based review of patients from the Alberta Cancer Registry between 1 January 2000 and 31 December 2015. Patients aged 18 years or older with biopsy-proven BTC (IHC, EHC, GBC, and AVT) were included. Patients without histopathology confirmed diagnosis or with pancreatic or hepatocellular tumors were excluded.

Data were collected via paper and electronic charts. Demographic data included age at diagnosis, sex, and race. Other data collected included risk factors for BTC (presence of primary sclerosing cholangitis, hepatolithiasis, or liver fluke infestation), symptoms at presentation, stage at diagnosis and site of metastasis if present, tumor marker levels (if performed), tumor location and stage, initial and subsequent treatment type (CT, radiotherapy), dosage and frequency of treatment, and survival outcomes. Data on resectability were collected and defined using procedure codes. If a procedure code was not recorded, a more in-depth chart review was performed, where possible, to determine resectability status based on available clinical records. Mean and standard deviations were reported for normally distributed continuous variables, and median and range were reported for non-normally distributed continuous variables. Time to event data for OS were analyzed using the Kaplan–Meier (KM) method and median estimates, and a 95% confidence interval was reported. The KM curves for OS by location, stage, type, and performance status were compared using log-rank tests. Our data were compared to published data in the literature. In our study, we defined OS as the time from pathologic diagnosis to death. Patients who were alive were considered censored and their last date of follow-up was used. The staging of tumors used the *Sixth Edition of the American Joint Committee on Cancer* (AJCC) staging criteria. Performance status was scored based on the Eastern Cooperative Oncology Group’s grading system from 0 to 5, with lower scores designating higher levels of functioning. Data were also collected on the location where patient care was instituted in the province based on zone within Alberta Health Services (AHS). AHS is Alberta’s health authority and is separated into 5 zones based on delivery of services. The largest population is in the Calgary zone, followed by the Edmonton and North, Central, and South Alberta (AB) zones.

## 3. Results

### 3.1. Patient Demographics

A total of 1719 patients with biopsy-proven BTCs were reviewed. We excluded 115 patients, of which 109 were excluded due to histopathology confirming hepatocellular or pancreatic origin or a lack of histopathology and an additional 6 datasets were removed due to duplicated or inconsistent information. Data from 1604 patients were included in our study. Table 1 summarizes the overall characteristics of our patient population at the time of diagnosis. Data are displayed by tumor location in Table 2. The median age was 68 years old (range of 19–99), and 47.8% were male.

By zone, the majority of patients were located in Calgary (n = 591, 36.8%), followed by Edmonton (*n* = 522, 32.5%), Central AB (*n* = 220, 13.7%), Northern AB (*n* = 157, 9.8%), and Southern AB (*n* = 112, 7.0%), respectively. Tumors were found more often in females than males in all zones. When known at diagnosis, the majority of patients in all zones had grade 2 tumors, stage 4 disease, and ECOG performance status 1, as was observed overall. Edmonton had the majority of tumors resected (n = 145, 28.0%), of which that were determined to be resectable at diagnosis. Calgary followed with 88 (14.9%) of 93, while resected tumors were found in 63 (28.6%) of 76 in Central AB, 41 (26.1%) of 42 in Northern AB, and 30 (26.8%) of 33 in Southern AB.

GBC was more prominent among females, whereas EHC and AVT had a male predominance. In each subtype, the majority of patients, when known, had a performance status of ECOG 1.

When reported, all tumor subtypes had majority grade 2 tumors. The majority of patients in the IHC, EHC, and GBC groups were stage 4 at diagnosis, with 249 (64.7%), 140 (35.3%), and 187 (35.3%) patients, respectively, having advanced, incurable disease.

Pathology confirmed adenocarcinoma NOS in 947 (59.2%) cases, cholangiocarcinoma in 307 (19.3%), and other types of neoplasms or in situ malignancy in the remaining 347 (22.2%). The GBC subtype was mostly adenocarcinoma NOS (*n* = 366, 69.2%), as was the case in the EHC subgroup (*n* = 232, 59.1%) and the AVT subgroup (*n* = 136, 55.9%).

### 3.2. Treatment

A total of 374 patients (23.3%) had tumors that were determined to be resectable at diagnosis. In contrast, 597 (37.2%) of tumors were determined to be unresectable. Resectability at diagnosis was unknown in the remaining 633 patients (39.5%). Of the resectable tumors, 333 (89.0%) went on to be resected. Of the unresectable tumors, 20 (3.4%) were eventually resected. Thirty-six patients (9.8%) with resections received adjuvant CT.

Of those patients with resected tumors, 259 (70.4%) did not receive any additional treatment. In those with unresected tumors, the majority received no treatment (73.8%). In those who did, 29 (4.3%) underwent radiation and 146 (21.9%) underwent some form of CT.

The majority of patients in our study (37.2%) did not receive any treatment at diagnosis, although eight of these cases (2.2%) went on to be resected. Of the patients, 551 (34.4%) underwent surgery alone for initial treatment, 289 (52.5%) of which had their tumors resected.

A total of 345 patients (21.5%) received CT, whereas 1259 (78.5%) did not. We know the specific CT regimen of 330 patients (20.6%) who received CT. Based on this available data, 3 (0.2%) patients received neoadjuvant, 56 (3.5%) received adjuvant CT, and 271 (16.9%) received palliative intent CT. The regimens used in the adjuvant setting were capecitabine, gemcitabine-based, and 5FU-based. For palliative intent CT, first-line regimens included gemcitabine alone, combination therapy with gemcitabine and cisplatin or gemcitabine and capecitabine, and capecitabine alone.

Of the patients, 81 (5.0%) received radiation therapy and 1523 (95.0%) did not. Whereas 33 received CT and radiation together (2.1%), 312 (19.5%) received CT alone. Meanwhile, 48 (3.0%) received radiation therapy alone and 1211 (75.5%) did not undergo either CT or radiation. It is worth noting that radiation therapy is not the standard of care in the treatment of cholangiocarcinoma in Alberta. We would expect these patients were receiving radiation for palliative treatment and symptom management.

### 3.3. Recurrence

GBCs had the lowest rate of tumor recurrence (*n* = 33, 6.2%), followed by BTC NOS (*n* = 0, 0%). EHC had the highest number of recurrences (*n* = 83, 21.1%), followed by IHC (*n* = 36, 9.4%).

### 3.4. Survival Outcomes

**Figure 1:**
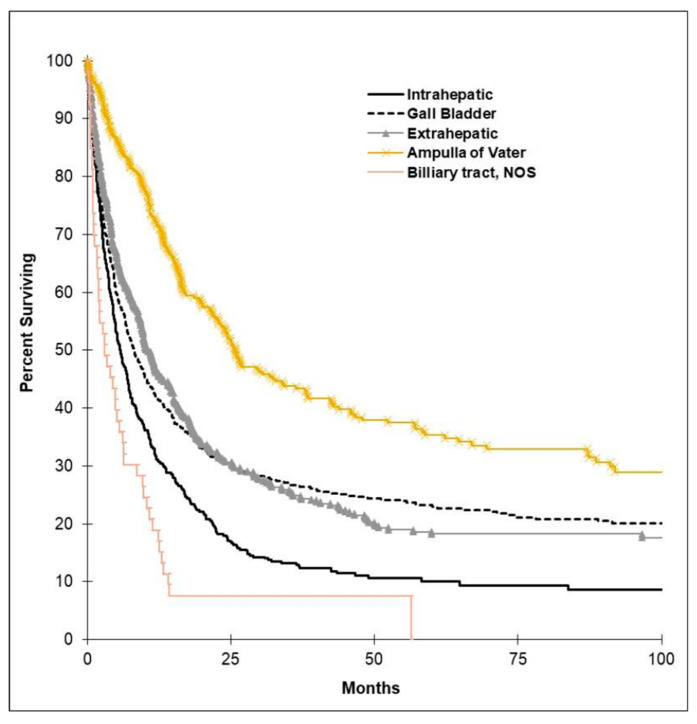
Kaplan–Meier curves depicting median overall survival (OS) by BTC subtype. The ampulla of Vater tumor (AVT) subtype had the longest median OS, with 25.7 months (95% confidence interval (CI), 19.7 to 31.7), followed by the extrahepatic cholangiocarcinoma (EHC) group with 10.3 months (95% CI, 8.5 to 12.1), gallbladder (GB) group with 8.1 months (95% CI, 6.6 to 9.7), and intrahepatic cholangiocarcinoma (IHC) group with 5.8 months (95% CI, 4.7 to 6.9). The NOS subtype had a median OS of 3.1 months (95% CI, 0.75 to 5.4).

**Figure 2:**
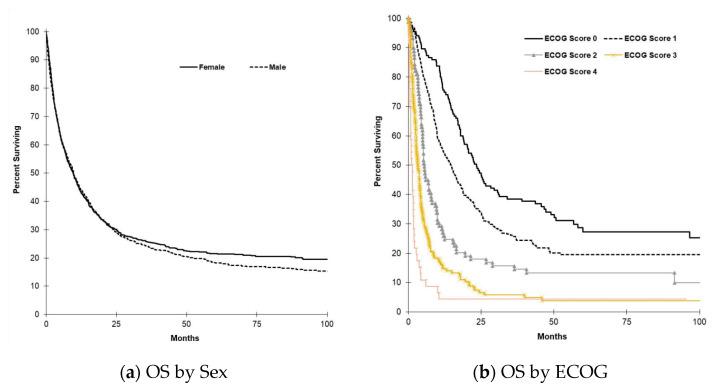

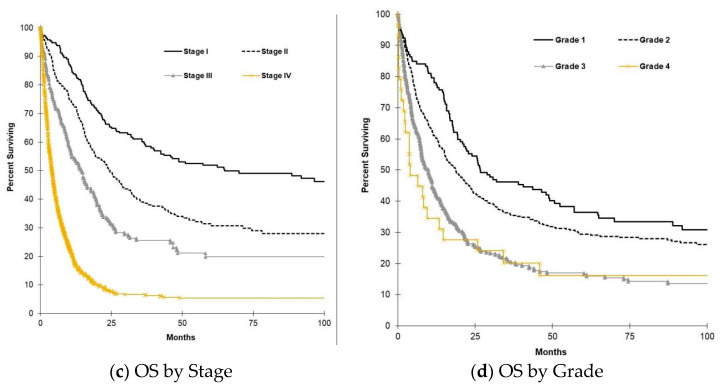
(**a**) Median OS by sex. The median OS in males for all subtypes was 9.8 months (95% CI, 8.4 to 11.2), while the median OS in females was 9.6 months (95% CI, 8.2 to 11.0; *P* = 0.306). (**b**) Median OS ECOG. Survival was higher among those with lower Eastern Cooperative Oncology Grou (ECOG) scores. An ECOG score of 0 had a median OS of 23.4 months (95% CI, 19.3 to 27.5), ECOG 1 of 14.5 months (95% CI, 11.7 to 17.3), ECOG 2 of 5.6 months (95% CI, 4.7 to 6.4), ECOG 3 of 3.3 months (95% CI, 2.4 to 4.1), and ECOG 4 of 1.1 months (95% CI, 0.7 to 1.6). (**c**) Median OS by stage. A similar trend was observed with stage. Patients classified as stage 1 had a median OS of 64.9 months 95% CI, 18.7 to 111.0), stage 2 of 24.2 months (95% CI, 18.2 to 30.3), stage 3 of 14.2 months (95% CI, 10.2 to 18.2), and stage 4 of 4.1 months (95% CI, 3.6 to 4.6). (**d**) Median OS by grade. The same trend was observed with overall survival when determined by grade at diagnosis. Those patients with grade 1 tumors at diagnosis had a median OS of 26.6 months (95% CI, 12.4 to 40.7), grade 2 of 18.7 months (95% CI, 15.1 to 22.3), grade 3 of 9.8 months (95% CI, 7.9 to 11.6), and grade 4 of 3.9 months (95% CI, 0.0 to 8.8).

**Figure 3:**
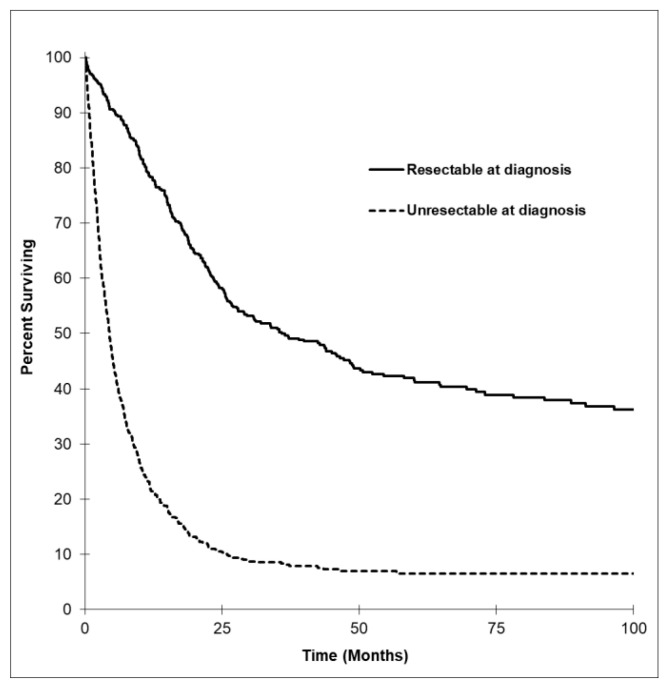
Kaplan–Meier survival curve based on resectability at diagnosis. Overall survival by resectability revealed the highest median OS in patients whose tumors were determined to be resectable at diagnosis with a median OS of 35.8 months (95% CI, 25.2 to 46.5) and was lower in those whose tumors were determined to be unresectable at diagnosis with a median OS of 4.4 months (95% CI, 3.9 to 5.0; *P* < 0.001).

Median OS by tumor subtype is shown in in Figure 1. The AVT subtype had the longest median OS, with 25.7 months (95% CI, 19.7 to 31.7), followed by EHC, GBC, and finally IHC. OS by ECOG, stage, and grade is illustrated in Figure 2. There was higher survival among those with better ECOG performance status and lower stage and grade of tumors. Figure 3 shows OS by tumor resectability. Those with tumors determined to be resectable at diagnosis had longer median OS of 35.8 months (95% CI, 25.2 to 46.5).

By zone, the highest OS was in the Calgary zone, with a median OS of 10.9 months (95% CI, 9.2 to 12.7), followed by Southern Alberta, Edmonton, Northern Alberta, and Central Alberta, with a median OS of 9.9 (95% CI, 3.8 to 16.0), 9.4 (95% CI, 7.7 to 11.1), 7.9 (95% CI, 4.7 to 11.1), and 7.8 months, respectively (95% CI, 3.8 to 16.0). OS for patients with advanced or metastatic disease (stage 3 or 4) was determined by zone. The highest OS in stage 3 tumors was 23.2 months (95% CI, 13.1 to 33.3) in Central Alberta, followed by 15.4 months in Calgary (95% CI, 8.5 to 22.3), 13.6 months (95% CI, 3.4 to 23.9) in Southern Alberta, 9.4 months (95% CI, 2.5 to 16.3) in Edmonton, and 7.9 months (95% CI, 0.4 to 15.5) in Northern Alberta. In stage 4 disease, the highest OS was 4.6 months (95% CI, 3.3 to 5.9) in the Calgary zone, followed by 4.4 months (95% CI, 3.7 to 5.1) in Edmonton, 3.6 months (95% CI, 2.8 to 4.4) in Northern Alberta, 3.1 months (95% CI, 2.3 to 3.8) in Central Alberta, and 2.2 months in the Southern Alberta zone (95% CI, 0.0 to 4.6).

**Figure 4:**
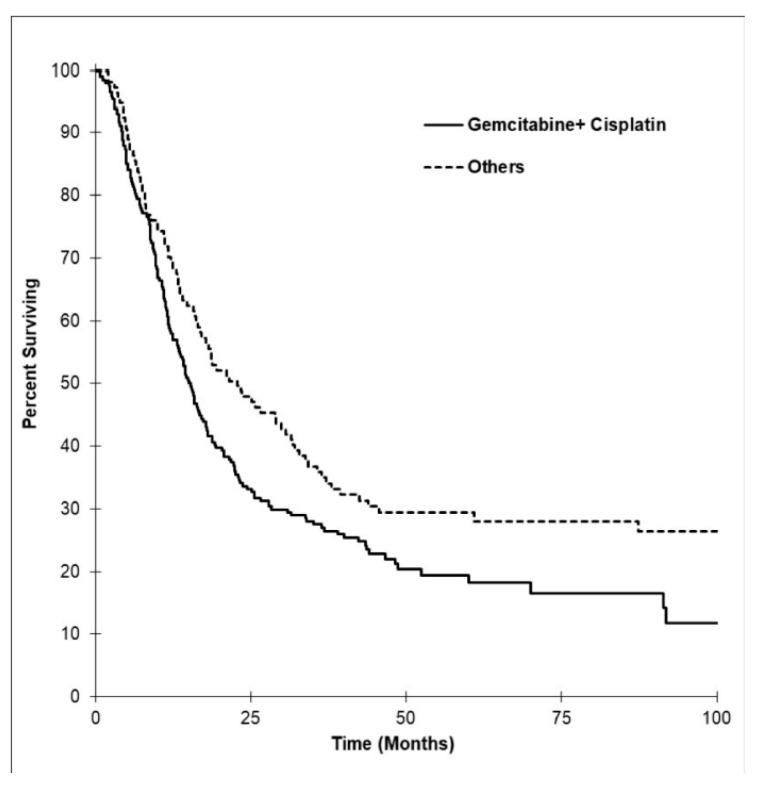
Kaplan–Meier survival curves by regimen. In the palliative-intent chemotherapy group, the gemcitabine cisplatin combination chemotherapy group included 212 patients. Compared to all other chemotherapy regimens, the median OS was 15.0 months (95% CI, 12.8 to 17.1) versus 11.0 months (95% CI, 7.0 to 15.0).

Overall survival was expectedly to be longer in the group that received adjuvant CT (36.4 months, 95% CI, 0.0 to 104.3) versus palliative-intent CT (12.5 months, 95% CI, 9.5 to 15.5). In those who had tumors resected, patients who received adjuvant CT had an OS of 45.7 months (95% CI, 0.0 to 129.6) compared with 39.0 months for those who did not (95% CI, 28.8 to 49.2). The most common first-line palliative regimen used was gemcitabine and cisplatin (*n* = 212, 80.9%), followed by gemcitabine alone (*n* = 22, 8.4%) and capecitabine with gemcitabine (*n* = 13, 5.0%). Median OS for the gemcitabine and cisplatin group compared to all other first-line regimens is reflected in Figure 4. OS for all patients who received first-line gemcitabine and cisplatin was 15.0 months (95% CI, 12.8 to 17.1), gemcitabine alone was 12.4 months (95% CI, 4.7 to 20.1), and capecitabine with gemcitabine was 6.9 months (95% CI, 2.2 to 11.7). When we analyzed only patients with advanced or metastatic disease (unresectable at diagnosis) who received palliative first-line gemcitabine and cisplatin, the OS was 11.8 months (95% CI, 9.3 to 14.3).

Median OS was explored pre- and post-publication of the 2010 randomized phase III clinical trial study by Valle et al. establishing cisplatin and gemcitabine as the standard therapy in advanced BTC. Subdivided by zone, median OS was higher prior to 2010 in Calgary (21.1 months, 95% CI 6.4 to 35.7 prior to 2010 versus 15.4 months, 95% CI 12.7 to 18.1 after 2010) and Southern Alberta zones (20.3, 95% CI 0.0 to 49.5 prior to 2010 versus 16.5 months, 95% CI 13.4 to 19.7), whereas in the other zones, median OS was higher after 2010 with 18.9 months (95% CI 11.8 to 26.0), 14.5 months (95% CI 6.4 to 22.6), and 23.8 months (95% CI 0.0 to 58.8) in the Edmonton, Central Alberta, and Northern Alberta zones, respectively. In patients with unresectable disease at diagnosis who received palliative intent after the 2010 publication, the highest median OS was 17.9 months in Northern Alberta (95% CI, 0.0 to 38.1), 14.2 months in Edmonton (95% CI, 10.0 to 18.5), 12.5 months in Southern Alberta (95% CI, 5.4 to 19.6), 9.8 months in Calgary (95% CI, 7.1 to 12.4), and 8.8 months in Central Alberta (95% CI, 6.1 to 11.5).

## 4. Discussion

BTCs carry a poor prognosis. In our study, OS decreased with more advanced stage and grade. Patients with AVT subtype had the best prognosis, while IHC had the worst.

There was minimal discrepancy in gender for all BTC subtypes, with the exception of the GBC subtype where prevalence was higher in females. This could reflect the higher prevalence of risk factors, such as cholelithiasis in North America among female patients, as is supported in the literature. Gallbladder cancer was the most common form of BTC among our patients, which is consistent with other demographic data available in the research.

Resectability should be a marker of disease aggressiveness as it indicates more advanced disease. Resectability is linked with better survival in the literature and our study also reflects this. Poorer OS was seen with more advanced tumor grading on histology, higher stage at diagnosis, and worse ECOG status at presentation.

The majority of patients who received palliative CT underwent the cisplatin and gemcitabine regimen, which has been the standard of care therapy since 2010. There was a small number of patients with unresected tumors who underwent CT, and looking at the data, the majority of these patients received CT after the standard of care was established. We noted better overall survival among those in the standard of care treatment group when compared with gemcitabine alone and with all other regimens combined.

Our patients with advanced or metastatic disease who received gemcitabine and cisplatin as first-line palliative CT had comparable OS to what was reported in the phase III trial by Valle et al. in 2010 (median OS of 11.8 versus 11.7 months). Our definition of OS differed from the original article, insofar as we defined OS from the date of pathologic diagnosis to the date of death, whereas the pivotal phase III trial defined it from the date of randomization to death. By zone, Calgary had the highest median OS overall compared to other zones in all comers. Calgary centers offered palliative CT regimens prior to the landmark phase III trial, establishing gemcitabine and cisplatin as the standard of care. Meanwhile, in Edmonton, palliative CT was generally only offered after 2010. When analyzed further, the data show that Calgary had higher OS prior to 2010 compared to after, as well as compared to Edmonton overall (p-values did not reach significance). When we narrowed the results to only advanced disease at diagnosis and palliative-intent CT after 2010, Edmonton had a higher survival rate compared to Calgary. Few centers offered CT to these patients prior to 2010.

Our study is a retrospective database review and thus has inherent limitations. Chart reviews lack a control group for comparison and have inherent selection and misclassification biases. With a lack of control group and randomization, causation cannot be determined. We are limited by the data available within the chart at the time of collection and the quality and quantity of data collected at the time of a clinical encounter. Further, we are subject to uncontrolled variables that may have impacted clinical interpretation and information at the time of data recording and coding. However, despite missing data, the observed trends in our data remain.

Our study reveals the impact of advanced disease and a patient’s overall functioning at diagnosis on outcomes as already evidenced in the literature and supports tumor resectability as a marker of overall survival. Our results reinforce the notion that surgical resection improves survival and therefore highlights the impact of early diagnosis on patient mortality. Moreover, it identifies the need for improved methods of tumor detection and therapy to target better rates of survival. This study provides insight into the demographic profile of BTC using a substantial population size. Along with other emerging areas of research, we can apply this knowledge both clinically and academically in an attempt to alter the landscape of BTC diagnosis, management, and prognosis. The current body of research on BTC offers a better understanding of the disease, prospective methods for earlier diagnosis, and suggests that more targeted therapies aimed at improving outcomes in this population are on the horizon, with randomized control trials likely to emerge over the next few years.

## Figures and Tables

**Table 1:** Patient characteristics (*n* = 1604).

Variable	Number of Patients (%)
Gender	
Male	766 (47.8%)
Female	838 (52.2%)
Median age in years (Range)	68 (19–99)
Tumor type	
Gallbladder	530 (33.0%)
Extrahepatic bile duct	394 (24.6%)
Intrahepatic bile duct	385 (24.0%)
Ampulla of vader	242 (15.1%)
Biliary tract NOS	53 (3.3%)
Tumor grade	
Grade 1	132 (8.2%)
Grade 2	443 (27.6%)
Grade 3	293 (18.3%)
Grade 4	29 (1.8%)
Unknown	707 (44.1%)
Lymphatic invasion	
Yes	106 (6.6%)
No	133 (8.3%)
Unknown	1365 (85.1%)
Perineural invasion	
Yes	142 (8.9%)
No	86 (5.4%)
Unknown	1376 (85.8%)
Disease stage	
Stage 0	24 (1.5%)
Stage 1	193 (12.0%)
Stage 2	220 (13.7%)
Stage 3	148 (9.2%)
Stage 4	632 (39.4%)
Stage unknown	387 (24.1%)
ECOG status	
ECOG 0	135 (8.4%)
ECOG 1	264 (16.5%)
ECOG 2	89 (5.5%)
ECOG 3	136 (8.5%)
ECOG 4	46 (2.9%)
ECOG unknown	934 (58.2%)
Resectability at diagnosis	
Resectable	374 (23.3%)
Not resectable	597 (37.2%)
Undetermined/Unknown	633 (39.5%)

**Table 2:** Patient and tumor characteristics by biliary tract cancer (BTC) subtype.

Variable	GBC	EHC	IHC	AVT	BTC NOS
Number	530	394	385	242	53
Male (%)	178 (33.6%)	228 (57.9%)	192 (49.9%)	139 (57.4%)	29 (54.7%)
Female (%)	352 (66.4%)	166 (42.1%)	193 (50.1%)	103 (42.6%)	24 (45.3%)
Age at diagnosis (y)	68 (26–99)	68 (26–95)	65 (19–89)	67 (31–91)	69 (29–88)
ECOG 0 (%)	7 (1.3%)	54 (13.7%)	60 (15.6%)	13 (5.4%)	1 (1.9%)
ECOG 1 (%)	27 (5.1%)	113 (28.7%)	104 (27.0%)	16 (6.6%)	4 (7.5%)
ECOG 2 (%)	5 (0.9%)	35 (8.9%)	48 (12.5%)	0 (0.0%)	1 (1.9%)
ECOG 3 (%)	12 (2.3%)	49 (12.4%)	66 (17.1%)	7 (2.9%)	2 (3.8%)
ECOG 4 (%)	2 (0.4%)	16 (4.1%)	27 (7.0%)	1 (0.4%)	0 (0.0%)
ECOG unknown (%)	477 (90.0%)	127 (32.2%)	80 (20.8%)	205 (84.7%)	45 (84.9%)
Grade 1 (%)	48 (9.1%)	34 (8.6%)	17 (4.4%)	32 (13.2%)	1 (1.9%)
Grade 2 (%)	139 (26.2%)	101 (25.2%)	100 (26.0%)	100 (41.3%)	3 (5.7%)
Grade 3 (%)	117 (22.1%)	46 (11.7%)	69 (17.9%)	58 (24.0%)	3 (5.7%)
Grade 4 (%)	13 (2.5%)	3 (0.8%)	8 (2.1%)	5 (2.1%)	0 (0.0%)
Grade unknown (%)	213 (40.2%)	210 (53.3%)	191 (49.6%)	47 (19.4%)	46 (86.8%)
Stage 0 (%)	13 (2.5%)	4 (1.0%)	0 (0.0%)	7 (2.9%)	0 (0.0%)
Stage 1 (%)	76 (14.3%)	46 (11.7%)	28 (7.3%)	43 (17.8%)	0 (0.0%)
Stage 2 (%)	73 (13.8%)	84 (21.3%)	16 (4.2%)	47 (19.4%)	0 (0.0%)
Stage 3 (%)	9 (1.7%)	44 (11.2%)	64 (16.6%)	30 (12.4%)	1 (1.9%)
Stage 4 (%)	187 (35.3%)	140 (35.3%)	249 (64.7%)	25 (10.3%)	31 (58.5%)
Stage unknown (%)	172 (32.5%)	76 (19.3%)	28 (7.3%)	90 (37.2%)	21 (39.6%)
Resectable at dx (%)	77 (14.5%)	174 (44.2%)	59 (15.3%)	64 (26.4%)	0 (0.0%)
Overall Resected (%)	99 (18.6%)	153 (38.8%)	52 (13.5%)	64 (26.4%)	0 (0.0%)

## Data Availability

The data presented in this study are available on request from the corresponding author. Data is not publicly available.

## Abbreviations

BTC	biliary tract cancers
CT	chemotherapy
OS	overall survival
ECOG	Eastern Cooperative Oncology Group
GBC	gallbladder cancer
IHC	intrahepatic cholangiocarcinoma
EHC	extrahepatic cholangiocarcinoma
AVT	ampulla of Vater tumors
